# A Dual Functional Diketopyrrolopyrrole‐Based Conjugated Polymer as Single Component Semiconducting Photoresist by Appending Azide Groups in the Side Chains

**DOI:** 10.1002/advs.202106087

**Published:** 2022-03-23

**Authors:** Chenying Gao, Dandan Shi, Cheng Li, Xiaobo Yu, Xisha Zhang, Zitong Liu, Guanxin Zhang, Deqing Zhang

**Affiliations:** ^1^ Beijing National Laboratory for Molecular Sciences Organic Solids Laboratory Institute of Chemistry Chinese Academy of Sciences Beijing 100190 China; ^2^ School of Chemical Sciences University of Chinese Academy of Sciences Beijing 100049 China; ^3^ State Key Laboratory of Applied Organic Chemistry (SKLAOC) College of Chemistry and Chemical Engineering Lanzhou University Lanzhou 730000 China

**Keywords:** conjugated polymers, cross‐linking, organic field‐effect transistors, photo‐patterning, semiconducting photoresists

## Abstract

Molecular systems that can function as photoresists are essential for the fabrication of flexible electronics through all‐photolithographic processes. Most of the reported molecular systems for photo‐patterning of polymeric semiconductors contain binary or multi‐components. In comparison, single component semiconducting photoresist is advantageous since it will circumvent the optimization of phase separation and ensure the patterned semiconducting thin films to be more uniform. In this paper, a single component semiconducting photoresist (PDPP4T‐N_3_) by incorporating azide groups into the branching alkyl chains of a diketopyrrolopyrrole‐based conjugated polymer is reported. The results reveal that i) the azide groups make the side chains to be photo‐cross‐linkable; ii) uniform patterns with size as small as 5 µm form under mild UV irradiation (365 nm, 85 mW cm^−2^) at ambient conditions; iii) such photo‐induced cross‐linking does not affect the inter‐chain packing; iv) benefiting from the single component feature, field‐effect transistors (FETs) with the individual patterned thin films display satisfactorily uniform performances with average charge mobility of 0.61 ± 0.10 cm^2^ V^–1^ s^–1^ and threshold voltage of 3.49 ± 1.43 V. These results offer a simple yet effective design strategy for high‐performance single component semiconducting photoresists, which hold great potentials for flexible electronics processed by all‐photolithography.

## Graphical abstract

A new diketopyrrolopyrrole‐based conjugated polymer as single component semiconducting photoresist has been developed by incorporating azide groups in the side chains. This conjugated polymer possesses excellent photo‐patterning property and high semiconducting performance simultaneously. Benefiting from the single component feature, the semiconducting performances of FETs with individual patterned thin films are satisfactorily uniform.

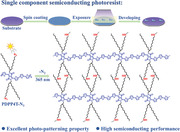

## 1 Introduction

Conjugated polymeric semiconductors have been regarded as the most promising candidates for flexible electronics^[^ ^1^ ^]^ due to their advantages of high charge mobility, excellent film‐forming property and soft feature.^[^ ^2^ ^]^ Although the semiconductor industry uses photolithography as the most widely used patterning technology with high‐precision, high‐reliability and high‐integration,^[^ ^3^ ^]^ it is challenging to process conjugated polymeric semiconductors into flexible circuits directly by photolithography.^[^ ^4^ ^]^ Compatible patterning of multi‐layers with different functions in single device is a precondition for the integration into circuits. However, the solubility of polymeric semiconductors makes it difficult to fabricate upper layers without dissolving the semiconducting layer during all‐solution processing. Therefore, developing facile immobilization and photo‐patterning strategies is in high demand for fabrication of polymeric semiconductor based flexible electronics by all‐photolithography.^[^ ^5^ ^]^

**Scheme 1:**
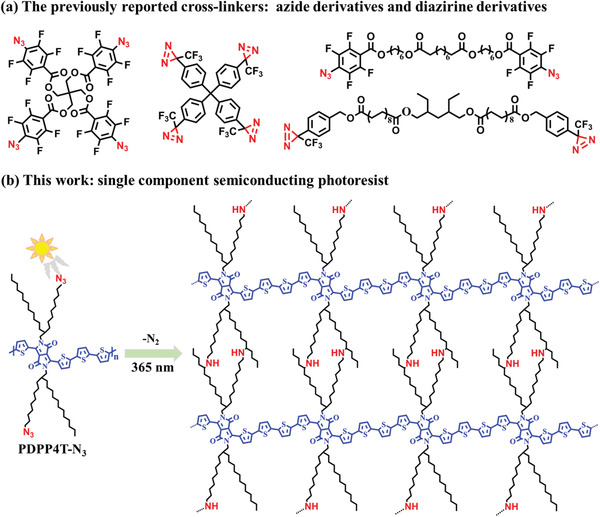
a) The chemical structures of the previously reported cross‐linkers for photo‐patterning of conjugated polymeric semiconductors. b) The chemical structure of single component semiconducting photoresist PDPP4T‐N_3_ in this work, and the illustration of the cross‐linking mechanism.

Photoresists that were used for immobilizing and patterning of semiconducting polymers were designed by utilizing photo‐chemical reactions. Indeed, compounds with azide^[^ ^1^, ^6^ ^]^ and diazirine^[^ ^7^ ^]^ groups (see **Scheme** **1**) were investigated as cross‐linkers to immobilize and photo‐pattern conjugated polymers. Azide and diazirine groups transform into their respective reactive nitrene and carbene species, which cross‐link the side chains of conjugated polymers, under UV light irradiation. As a consequence, the blending of conjugated polymers with cross‐linkers has been successfully utilized for photo‐patterning of polymeric semiconductors with good resolution. The resulting patterned polymeric semiconducting thin films show good resistance to organic solvents. Bao, Kim, and their co‐workers have utilized these cross‐linkers successfully to fabricate all‐solution‐processed integrated devices.^[^ ^1^, ^6^ ^]^ Some of us have recently reported a four‐armed cross‐linker with four diazirines for efficient photo‐patterning of *p*‐, *n*‐, and ambipolar polymeric semiconductors.^[^ ^7b^ ^]^ In these cases, controlling the miscibility between cross‐linkers and polymeric semiconductors is required to ensure the uniformity and repeatability of photo‐patterning.^[^ ^8^ ^]^

Alternative strategies for photo‐patterning of polymeric semiconductors are based on the blending of semiconducting polymers with photo‐cross‐linkable insulating additives, such as SU8,^[^ ^9^ ^]^ PCell^[^ ^9^ ^]^ and poly(vinyl cinnamate),^[^ ^10^ ^]^ which can be cross‐linked to form an insulating polymer matrix. Thus, the phase‐separated nanofibers of polymeric semiconductors are immobilized by the cross‐linked insulating polymer matrix after exposure to UV light, and photo‐patterning is achieved with the photomasks. Wei and co‐workers have recently patterned semiconducting thin films and fabricated field‐effect transistors (FETs) array with the quaternary blend of a diketopyrrolopyrrole (DPP) polymer, cross‐linkable tris[2‐(acryloyloxy)ethyl] isocyanurate, a radical photo‐initiator and a thiol additive.^[^ ^11^ ^]^ For this immobilization and photo‐patterning strategy, the polymeric semiconductors need to be blended with multi‐component additives, and the side chains of polymeric semiconductors do not participate in any photo‐chemical reaction.

Incorporation of photo‐cleavable groups into the side chains has also been investigated for photo‐patterning of polymeric semiconductors.^[^ ^12^ ^]^ In this case, the removal of photo‐cleavable groups under light irradiation leads to the reduction of the solubility of polymer thin film. Consequently, the semiconducting polymer thin film can be patterned similarly as ordinary photoresists using standard lithographic techniques. But, the photo‐reaction products may be left in the semiconducting thin films. Apart from photo‐cleavable groups, reactive groups such as oxetane,^[^ ^13^ ^]^ for which the ring‐opening reaction occurs in the presence of photo‐initiator under UV light irradiation, was also introduced to the side chains of polymeric semiconductors for photo‐patterning. It is noted that incorporation of photo‐reactive groups in the side chains of conjugated polymers stabilizes the morphologies of active layers of organic solar cells (OSCs)^[^ ^14^ ^]^ and organic light‐emitting diode (OLEDs)^[^ ^15^ ^]^ and thus improve the device stabilities.^[^ ^16^ ^]^

As discussed above, most of the reported photoresists involve binary or multi‐components. The phase separation of binary or multi‐component could disturb the uniformity, repeatability and precision of patterns of semiconducting thin films. To address this limitation, it is critical to develop high performance single component semiconducting photoresists. In this paper, we report a new single component semiconducting photoresist by appending the azide groups in the side chains of DPP based conjugated polymer (PDPP4T‐N_3_ in Scheme 1). The molecular design is based on the following considerations: i) DPP‐based conjugated D‐A polymers are known as semiconductors with high charge mobilities;^[^ ^2^, ^17^ ^]^ ii) azide groups in PDPP4T‐N_3_ can be transformed into reactive nitrene species, resulting in the cross‐linking of side alkyl chains,^[^ ^16^, ^18^ ^]^ upon light irradiation. The polymer chains of PDPP4T‐N_3_ are cross‐linked after UV light irradiation and consequently their solubility is expected to be largely reduced. Accordingly, photo‐patterned PDPP4T‐N_3_ thin films are easily formed with the aid of photomask after treatment of the developer; iii) the azide groups are incorporated into the branching alkyl chains, which will induce large solubility difference between the cross‐linked and the pristine polymer. As a result, well‐resolved patterned thin films of PDPP4T‐N_3_ can be obtained. The results reveal that PDPP4T‐N_3_ is a semiconducting photoresist. Different photo‐patterns with a resolution of 5 µm were successfully generated after irradiation of the PDPP4T‐N_3_ with UV light (365 nm, 85 mW cm^−2^) for 400 s. The patterned stripes of PDPP4T‐N_3_ were individually utilized to fabricate FETs and the devices show good performance with average charge mobility of 0.61 ± 0.10 cm^2^ V^–1^ s^–1^. Moreover, the devices exhibit satisfactory uniformity in terms of charge mobility and threshold voltage. The cross‐linked thin films of PDPP4T‐N_3_ are resistant to organic solvents, and the *n*‐type semiconducting polymer F_4_BDOPV‐2T (see Figure 5 and Figure S1a, Supporting Information) can be fabricated directly by spin coating without using orthogonal solvent and the corresponding *p*‐ and *n*‐channels were used to construct an inverter with a gain value of 68.

## 2 Results and Discussions

### 2.1 Synthesis and Characterization

**Scheme 2:**
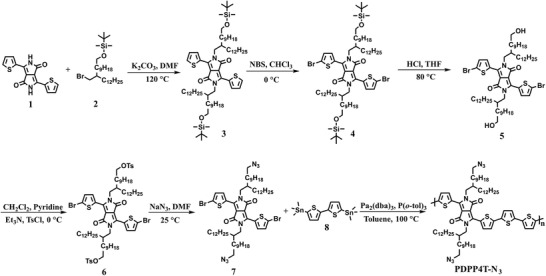
The synthetic route for PDPP4T‐N_3_.

The synthesis of PDPP4T‐N_3_ is outlined in **Scheme** **2**. Firstly, the alkylation of **1** with **2** with a branching chain capped with *t*‐butyldimethylsiloxy (TBDMS) yielded **3** in 30% yield. Bromination of **3** was accomplished with *N*‐bromosuccinimide (NBS). After deprotection of TBDMS, sulfonylation and reaction with NaN_3_, the monomer **7** was obtained in a total yield of 27%. PDPP4T‐N_3_ was prepared by following the conventional Stille polymerization procedure, the polymerization reaction temperature was kept at 100 °C. All operations involving azide‐containing species were kept away from UV light. The synthetic and purification details are provided in the Supporting Information.

The number‐average molecular weight (*M*_n_) and the dispersity (*Ð*) of PDPP4T‐N_3_ were 28.0 kDa and 1.7, respectively, according to gel permeation chromatography (GPC, eluent *o*‐DCB at 140 °C, Figure S2, Supporting Information). Based on its thin film absorption spectrum and cyclic voltmmogram (see Figure S3, Supporting Information), the optical bandgap, HOMO and LUMO energy levels of PDPP4T‐N_3_ were estimated to be 1.28, −5.27, and −3.38 eV, respectively, being close to those of PDPP4T (see Figure S1b, Supporting Information) reported previously.^[^ ^17a^ ^]^ Figure S4 (Supporting Information) shows the FT‐IR spectrum and the stretching signal at 2094 cm^–1^, due to the azide groups in the side chains, was detected. Thermogravimetric analysis data show that the weight loss of PDPP4T‐N_3_ reached 4.58% in the temperature range of 170–260 °C under nitrogen atmosphere (Figure S5, Supporting Information), consistent with the theoretical weight reduction caused by loss of N_2_.

### 2.2 Cross‐Linking of the Polymeric Side Chains

**Figure 1:**
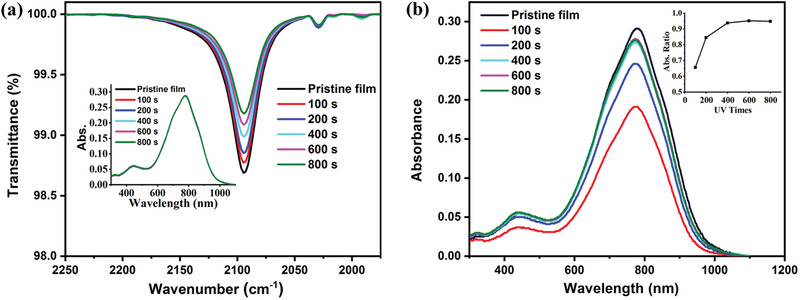
a) The variation of FT‐IR spectra of thin film of PDPP4T‐N_3_ after UV irradiation (365 nm, 85 mW cm^−2^) for different times. The inset shows the variation of thin film absorption spectra after UV irradiation (365 nm, 85 mW cm^−2^) for different times. b) The variation of thin film absorption spectra of PDPP4T‐N_3_ after UV irradiation (365 nm, 85 mW cm^−2^) for different times followed by being soaked in chloroform for 20 s. The inset shows the variation of the ratio of absorbance at 780 nm by comparing with those of the respective pristine films.

Next, we show that the cross‐linking of side chains of PDPP4T‐N_3_ upon UV light irradiation. As shown in **Figure** **1**a, the FT‐IR stretching signal at 2094 cm^–1^ decreased gradually after the thin film of PDPP4T‐N_3_ was irradiated with 365 nm light attributed to the transformation of azide into nitrenes resulting in the C–H insertion and cross‐linking of side alkyl chains.^[^ ^6^, ^18^ ^]^ Figure 1b shows the absorption spectra of the thin film of PDPP4T‐N_3_, which was exposed to UV light for different times, followed by thoroughly rinsing with CHCl_3_ (developer). These results reveal that i) the reactive nitrenes do not react with the conjugated backbone as the absorption spectrum of the cross‐linked polymer (*λ*_max_ 780 nm) is the same as that of the pristine thin film (inset of Figure 1a); ii) thin film of PDPP4T‐N_3_ is immobilized by the cross‐linking with nitrenes. The pristine film of PDPP4T‐N_3_ is completely dissolved by CHCl_3_, while the retention of the absorbance of DPP backbone at 780 nm increased by prolonging the irradiation time. The retention ratio of absorbance was more than 95% after the film was irradiated with 365 nm light for 400 s.

### 2.3 Photo‐Patterning of Polymeric Semiconductor

**Figure 2:**
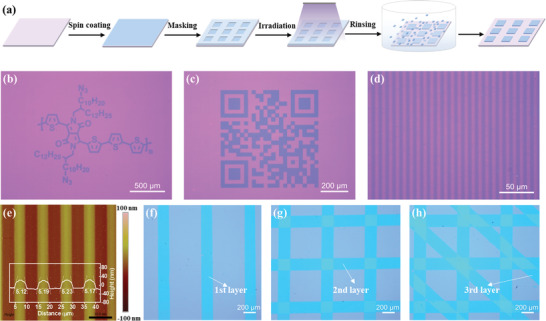
a) Illustration of photo‐patterning process of PDPP4T‐N_3_. b–d) Optical microscope images of patterns of PDPP4T‐N_3_ with different shapes. e) AFM height image (50 × 50 µm) and the corresponding height profile of the striped patterns in (d). f‐h) Optical microscope images of step‐by‐step patterning of three layers of PDPP4T‐N_3_. The exposure time was 400 s with a power density of 85 mW cm^−2^, and the development time was 20 s for all patterns.

Photo cross‐linking occurs with PDPP4T‐N_3_ and allows the patterns to be transferred from mask to the substrate, making PDPP4T‐N_3_ a negative semiconducting photoresist. The photo‐patterning of PDPP4T‐N_3_ was carried out at ambient conditions. Thin films of PDPP4T‐N_3_ with thicknesses of ≈40 nm were fabricated on SiO_2_/Si substrates by spin‐coating a solution of PDPP4T‐N_3_ in chloroform (**Figure** **2**a). Then, masks with different transparent patterns were placed on the substrates. After exposure to 365 nm UV light with a power density of 85 mW cm^−2^ for 400 s, the substrates were soaked in chloroform for 20 s to remove thin films in the unexposed area and the respective patterns appeared accordingly.

Figure 2b–d shows the optical microscope images of the representative patterns such as chemical structure, and two‐dimensional code, which can be precisely transferred to the substrates. Especially, a striped pattern with uniform thickness of ≈40 nm and width as small as 5 µm was easily acquired, and the width deviation was less than 5% (Figure 2e). Moreover, patterns with multilayers were also successfully fabricated through a step‐by‐step patterning approach (see Figure 2f‐h). Due to the robust cross‐linked network, the interfaces between different layers were distinct. Therefore, the efficient photo‐induced cross‐linking of side chains will greatly expand the processability of PDPP4T‐N_3_ as a semiconducting photoresist for all‐photolithographic flexible electronics.

### 2.4 Morphology and Inter‐Chain Packing of the Patterned Thin Films

**Figure 3:**
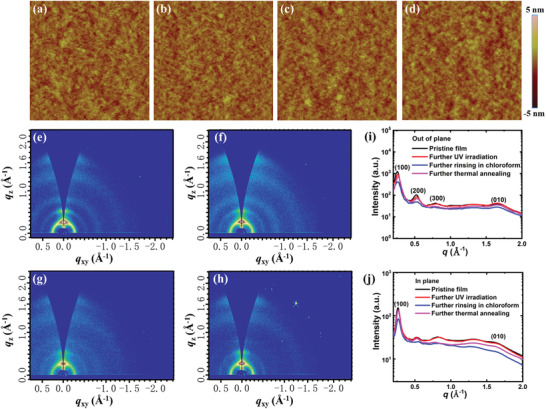
AFM height images (1 × 1 µm) and GIWAXS patterns of PDPP4T‐N_3_ at different steps during the photo‐patterning processes and after thermal annealing. a,e) The pristine films. b,f) The films after exposure to 365 nm UV light with a power density of 85 mW cm^−2^ for 400 s. c,g) The cross‐linked films after further being soaked in chloroforms for 20 s. d,h) The rinsed films after further thermal annealing at 160 °C for 10 min. i,j) The corresponding *out‐of‐plane* and *in‐plane* cuts of GIWAXS patterns.

Both the pristine and the photo‐patterned films were characterized by atomic force microscopy (AFM) and two‐dimensional grazing‐incidence wide‐angle X‐ray scattering (GIWAXS) measurements. As shown in **Figure** **3**a–d, all films display similar fibril‐like structures and roughness. This shows that the surface morphology of PDPP4T‐N_3_ is not affected by irradiation and the surface morphology of the cross‐linked PDPP4T‐N_3_ is robust against solvent and heating.

Figure 3e–h shows the GIWAXS patterns of pristine films and the films during photo‐patterning processes. Like most of the reported DPP‐based conjugated polymers, the pristine thin film of PDPP4T‐N_3_ shows semi‐crystalline feature, and both lamellae stacking of alkyl chains and inter‐chain *π*–*π* stacking signals were detected. The detailed *q* values and the corresponding *d*‐spacing values of the (100) and (010) diffraction peaks at different steps during the photo‐patterning processes and after further thermal annealing are summarized in Table S1 (Supporting Information). The intensity of (100) diffraction peaks decreases slightly and the peak positions of *q* values are shifted from 0.26 to 0.27 Å^–1^ after UV light irradiation. This is understandable because the conversion of azide groups and the resulting cross‐linking of side chains can shorten the lamellar spacing of the polymer chains and slightly affect the packing order of polymer chains. The intensity of (h00) diffraction peaks decreases obviously after thin films were rinsed with chloroform, which can be probably attributed to the swelling of the cross‐linked polymer chains by chloroform and thus the packing order of polymer chains is affected. It is worth noting that the intensity of all diffraction peaks at both *out‐of‐plane* and *in‐plane* directions are significantly enhanced after annealing at 160 °C for 10 min. As a result, the inter‐chain packing within the patterned thin film of PDPP4T‐N_3_ was not changed noticeably after thermal annealing.

### 2.5 Charge Transport Properties of the Patterned FETs

**Figure 4:**
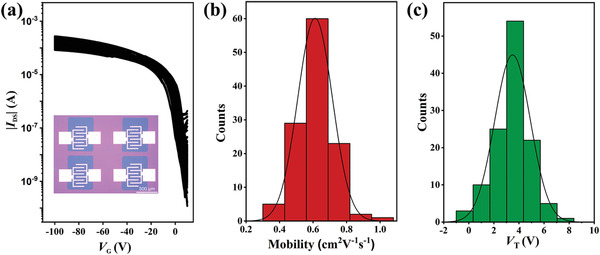
a) Transfer curves of 120 independent patterned FET devices. The inset shows the optical microscope images of the patterned devices. b,c) The corresponding distributions of mobilities, and threshold voltages. The exposure time was 400 s with a power density of 85 mW cm^−2^ and the development time was 20 s for all patterns. All patterned devices were annealed at 160 °C for 10 min before measurements.

FETs with bottom‐gate/bottom‐contact (BGBC) configuration were fabricated from PDPP4T‐N_3_ (for details, see the Supporting Information). Briefly, thin films of PDPP4T‐N_3_ with thicknesses of ≈40 nm were prepared by spin coating a solution of PDPP4T‐N_3_ in chloroform onto the octadecyltrichlorosilane (OTS) modified SiO_2_/Si substrates with gold as drain and source electrodes. Then, masks with transparent rectangular patterns were placed on the films. After UV light (365 nm, 85 mW cm^−2^) irradiation for 400 s and developing with CHCl_3_, rectangular patterns with width and length of 600 and 800 µm, respectively, were formed on the top of electrodes as shown in the inset of **Figure** **4**a. In total, 120 FETs with different individual patterned thin films were fabricated and measured.

Figure 4a and Figure S6 (Supporting Information) show the transfer curves of 120 FETs and the representative output curves. As expected, these devices show typical *p*‐type semiconducting property. Charge mobilities and threshold voltages of 120 devices were extracted in the conventional way. Figure 4b,c shows the distributions of charge mobilities and threshold voltages. The average charge mobility is 0.61 ± 0.10 cm^2^ V^–1^ s^–1^, and the average *V*_th_ is 3.49 ± 1.43 V. The distributions of both *μ*_h_ and *V*_th_ are relatively narrow. Therefore, the performance of these 120 devices is satisfactorily uniform. As PDPP4T‐N_3_ functions both as semiconductor and photoresist, consequently the photo‐controllable cross‐linking is uniform.

For comparison, the BGBC device with the pristine thin film of PDPP4T‐N_3_ was also fabricated in the same way as for those with the patterned thin films (Figure S7, Supporting Information). The average charge mobility was measured to be 1.06 ± 0.32 cm^2^ V^–1^ s^–1^, higher than those of FETs with the patterned thin films, although the pristine and the patterned thin films display rather similar GIWAXS patterns and thin film morphology. The lower charge mobility of the patterned thin films is likely due to the presence of amino groups in the side chains, formed during the cross‐linking reactions (see Scheme 1) and they are potential traps of hole carriers. Figure S4 (Supporting Information) shows the FT‐IR spectra of the pristine film of PDPP4T‐N_3_ and the film after UV irradiation for 400 s. After UV irradiation, the intensity of the azide groups at 2094 cm^–1^ decreased, and simultaneously the intensity at 3200–3400 cm^–1^ increased, indicating the formation of amino groups after UV irradiation.^[^ ^18^ ^]^ The presence of amino groups in the cross‐linked thin films increases the interfacial trap state density (*N*_int_) (see Table S2, Supporting Information), which was calculated from the subthreshold slope.^[^ ^19^ ^]^ Consequently, it is understandable that hole mobilities of FETs with the cross‐linked thin films of PDPP4T‐N_3_ decreased gradually by prolonging the UV light irradiation time as shown in Figure S8 (Supporting Information).

**Figure 5:**
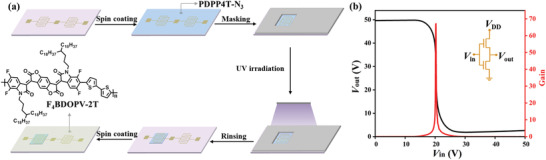
a) Illustration of the complementary‐like inverter fabrication; the chemical structure of F_4_BDOPV‐2T. b) The corresponding voltage transfer and gain characteristics measured under nitrogen atmosphere, *V*_DD_ = 50 V. The inset shows the circuit diagram.

The excellent patterning property and high semiconducting performance of PDPP4T‐N_3_ prompted us to fabricate logic gates entirely through solution processes. Logic inverter as one of the basic building blocks, plays important roles in integrated circuit.^[^ ^20^ ^]^ Solution processed organic complementary‐like inverters are usually fabricated by two identical FETs based on one ambipolar organic semiconductor.^[^ ^21^ ^]^ However, the major obstacle is that the highly balanced ambipolar organic semiconductor with outstanding semiconducting performance is rare.^[^ ^2d^ ^]^ Benefiting from the robust cross‐linked network of PDPP4T‐N_3_, it is easy to deposit the second component without an orthogonal solvent. To this end, we present here the use of patterned thin films of PDPP4T‐N_3_ and thin film of another *n*‐type polymeric semiconductor (F_4_BDOPV‐2T, see **Figure** **5**a and Figure S10, Supporting Information) to construct a complementary‐like inverter. PDPP4T‐N_3_ was first patterned on the pre‐prepared SiO_2_/Si substrates with gold as drain and source electrodes. Then, thin film of F_4_BDOPV‐2T was directly spin coated from chloroform. As shown in Figure 5b, the voltage transfer curve displays a distinct switching‐action around 20 V with a gain value of 68. The results demonstrate that the single component semiconducting photoresist such as PDPP4T‐N_3_ will make it much easier to fabricate inverters by the free selection of semiconductors entirely through solution processes.

## 3 Conclusion

We report a DPP‐based conjugated D‐A polymer PDPP4T‐N_3_ which can function as both high charge mobility semiconductor and negative photoresist simultaneously by appending azide groups in the branching alkyl chains. The incorporation of azide groups in the side alkyl chains enables the polymer chains of PDPP4T‐N_3_ to be photo‐cross‐linkable though the formation of reactive species of nitrene. Such photo‐induced cross‐linking renders the significant reduction of the polymer solubility, but it does not affect the absorbance spectra and energy level of DPP backbone. By utilizing this feature, photo‐patterning of thin film of PDPP4T‐N_3_ can be easily achieved with appropriate photomasks. In particular, regularly striped patterns with size as small as 5.0 µm can be photo‐generated without destroying the *π*–*π* stacking of conjugated main chains. The semiconducting performances of FETs with individual patterned thin films of PDPP4T‐N_3_ are satisfactorily uniform with average charge mobility of 0.61 ± 0.10 cm^2^ V^–1^ s^–1^ and threshold voltage of 3.49 ± 1.43 V. The advantage of such photo‐patterning strategy is further demonstrated by the easy construction of an inverter entirely through solution processes. It is expected that PDPP4T‐N_3_ and its analogues are useful as single component semiconducting photoresists for photo‐pattering of other polymeric semiconductors and hold great potentials for organic flexible electronics processed by all‐photolithography.

## Conflict of Interest

The authors declare no conflict of interest.

## Supporting information

Supporting Information

## Data Availability

The data that support the findings of this study are available in the supplementary material of this article.
