# Supplementary material for: A Dual Functional Diketopyrrolopyrrole‐Based Conjugated Polymer as Single Component Semiconducting Photoresist by Appending Azide Groups in the Side Chains
Source: Adv Sci (Weinh). 2022 Mar 23;9(15):2106087. doi: 10.1002/advs.202106087 (PMC9130897; doi:10.1002/advs.202106087)
Supplement: Supplementary file 1 — Supporting Information [file ADVS-9-2106087-s001.pdf]

ADVANCED SCIENCE
Open Access

Supporting Information

for *Adv. Sci.*, DOI 10.1002/advs.202106087

A Dual Functional Diketopyrrolopyrrole-Based Conjugated Polymer as Single Component Semiconducting Photoresist by Appending Azide Groups in the Side Chains

Chenying Gao, Dandan Shi, Cheng Li, Xiaobo Yu, Xisha Zhang, Zitong Liu, Guanxin Zhang and Deqing Zhang**

Supporting Information

A Dual Functional Diketopyrrolopyrrole-based Conjugated Polymer as Single Component Semiconducting Photoresist by Appending Azide Groups in the Side Chains

Chenying Gao, Dandan Shi, Cheng Li, Xiaobo Yu, Xisha Zhang, Zitong Liu, Guanxin Zhang, and Deqing Zhang**

Table of Contents

1. Materials and measurements
2. Synthesis of **PDPP4T-N$_3$**
3. The chemical structure of **F$_4$BDOPV-2T** and **PDPP4T**
4. Gel Permeation Chromatography (GPC) of **PDPP4T-N$_3$**
5. Absorption spectrum and cyclic voltammogram
6. FT-IR spectra
7. Thermogravimetric analysis (TGA) of **PDPP4T** and **PDPP4T-N$_3$**
8. Parameters of the GIWAXS patterns
9. Output curve of FETs with patterned thin films of **PDPP4T-N$_3$**
10. Performance data of FETs with pristine thin films of **PDPP4T-N$_3$**
11. Optical microscope image of inverter devices
12. NMR spectra
13. References

1. Materials and measurements

All chemicals were purchased from commercial suppliers and used without further purification unless otherwise specified. Compounds **1** and **2** were purchased from Woerjiming (Beijing) Technology Development Institute, and Jiujiang Design Optoelectronic Materials Co., Ltd., respectively. **F_4BDOPV-2T** was purchased from Beijing Hwrkchemical Co., Ltd., with number-average molecular weight (M_n) of 16.8 kDa and dispersity ($Đ$) of 3.7. **PDPP4T** was synthesized according to the procedures reported in the literature[S1] with number-average molecular weight (M_n) of 40.3 kDa and dispersity ($Đ$) of 1.4. All operations involving azide containing species were kept away from UV light.

^{1}H NMR, ^{13}C NMR spectra in solution and the solid state ^{13}C NMR spectrum were recorded at a Bruker AVANCE III 400 MHz spectrometer. High-temperature ^{1}H NMR, ^{13}C NMR spectra were recorded at a Bruker Avance III 500WB NMR Spectrometer. Matrix-Assisted Laser Desorption/Ionization Time of Flight (MALDI-TOF) mass spectra were collected on a Bruker Solarix 9.4T FT-ICR mass spectrometer. Elemental analyses were performed on a Thermo Flash Smart instrument. Gel permeation chromatography (GPC) was performed on a Agilent PL-GPC 220 instrument using *o*-dichlorobenzene as an eluent (1.0 mL/min) at 140 °C. Polystyrene was utilized as the calibration standard, and **PDPP4T-N_3** was dissolved in *o*-dichlorobenzene and the concentration was 1 mg/mL.

Thermogravimetric analysis (TGA) analyses were carried on a PerkinElmer TGA 8000 Thermogravimetric Analyzer instrument under N_2 at a heating rate of 10 °C/min from 50 °C to 550 °C. UV-vis-NIR absorption spectra were collected on a HITACHI UH4150 UV-Vis spectrophotometer. **PDPP4T-N_3** was dissolved in $CHCl_3$ and the concentration was 5 mg/mL. Thin film was fabricated by spin coating on quartz substrates. Fourier transform infrared spectra were recorded on a Bruker TENSOR-27. Cyclic voltametric (CV) measurements were carried out in a three-electrode cell by using glassy carbon as the working electrode, Pt as auxiliary electrode, and Ag/AgCl (saturated aqueous solution of KCl) as reference electrode on a computer-controlled CHI660C instrument at room temperature; the scan rate was 100 mV/s, and *n*-Bu_4NPF_6 (0.1 mol/L in acetonitrile) was used as the supporting electrolyte. For calibration, the redox potential of ferrocene/ferrocenium (Fc/Fc^+) was measured under the same conditions. HOMO and LUMO energy levels of this conjugated polymer was estimated with the following equations: HOMO = $-(E_{ox}^{onset} + 4.8)$ eV, LUMO = $-(E_{red}^{onset} + 4.8)$ eV.

Atomic force microscopy (AFM) images were recorded using a Digital Instruments Nano scope IIIa multimode atomic force microscope in tapping mode under ambient conditions. Optical microscope images were recorded using a Leica-DM4M microscope. Two-dimensional grazing-incidence wide-angle X-ray scattering (GIWAXS) measurements were

conducted on a Xenocs-SAXS/WAXS system with X-ray wavelength of 1.5418 Å. The film samples were irradiated at a fixed angle of 0.2°. A DSX-UVP60 UV LED Curing System was used as light source for the photo-patterning, and the light intensity was detected by a CEL-NP2000 optical power meter.

OFET and inverter devices were fabricated on a commercial Si/SiO_2/Au substrate purchased from First MEMS Co. Ltd. A heavily *n*-doped Si wafer with a SiO_2 layer of 300 nm served as the gate electrode and dielectric layer, respectively. The Ti (2 nm)/Au (28 nm) source-drain electrodes were sputtered and patterned by a lift-off technique. Before deposition of the organic semiconductor, the gate dielectrics were treated with octadecyltrichlorosilane (OTS) in a vacuum oven at 120 °C, forming an OTS self-assembled monolayer, according to the reported method.[S2] Then, the substrates were washed with $CHCl_3$, hexane and isopropanol, sequentially. Polymer thin films were spin coated on the substrate with a thickness of around 30 - 50 nm. The devices were thermally annealed at 160 °C for 10 min before measurement (in air for hole mobility measurement, while in a glovebox filled with N_2 for inverter measurement). The devices were measured on a Keithley 4200 SCS semiconductor parameter analyzer at room temperature. The channel width was 1400 μm, and the channel length was 50 μm. The calculations of mobilities, threshold voltages, on-off current ratios were carried out according to our previous report.[S2]

2. Synthesis of PDPP4T-N_3

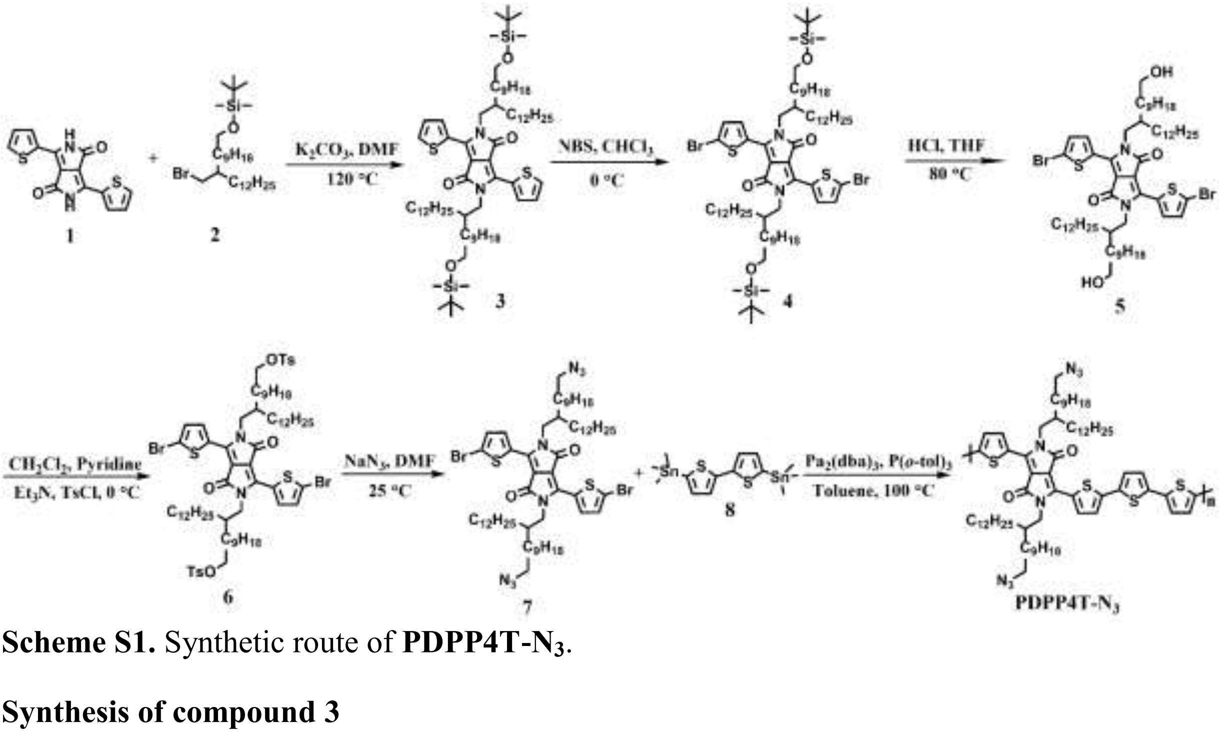

Scheme S1. Synthetic route of **PDPP4T-N_3**.

Synthesis of compound 3

Compound **1** (3,6-di(thiophen-2-yl)-2,5-dihydropyrrolo[3,4-c] pyrrole-1,4-dione) (2.00 g,

6.66 mmol) and K_2CO_3 (4.60 g, 33.17 mmol) were added to a 100 mL Schlenk tube, and 40 mL of dry *N, N*-dimethylformamide was injected by syringe under nitrogen atmosphere. The reaction was heated to 120 °C for 30 min. Then compound **2** (((11-(bromomethyl) tricosyl) oxy) (tert-butyl) dimethylsilane) (8.39 g, 15.32 mmol) was slowly added dropwise by a syringe. After stirred for 7 h at 110 °C, the mixture was extracted by dichloromethane and washed with saturated aqueous solution of NaCl for 3 times. After removal of solvents, the product was purified by silica gel column chromatography with petroleum ether (b.p. 60 - 90 °C) and dichloromethane (3:1, *v*/*v*) as eluents to give compound **3** as a red-brown solid (2.47 g, 30.0%). ^{1}H NMR (400 MHz, CH_2Cl_2) : δ = 8.82 (d, J = 3.6 Hz, 2H), 7.66 (d, J = 4.8 Hz, 2H), 7.28 (t, J = 4.0 Hz, 2H), 4.00 (d, J = 7.6 Hz, 4H), 3.58 (t, J = 6.8 Hz, 4H), 1.87(m, 2H), 1.49-1.44 (m, 4H), 1.30-1.22 (m, 76H), 0.91-0.86 (m, 24H), 0.04 (s, 12H).^{13}C NMR (100 MHz, $CDCl_3$): δ = 161.75, 140.42, 135.21, 130.43, 129.86, 128.39, 107.96, 77.34, 77.23, 77.02, 76.71, 63.34, 46.23, 37.77, 32.92, 31.94, 31.24, 31.19, 30.06, 30.03, 29.71, 29.69, 29.66, 29.62, 29.57, 29.48, 29.38, 26.24, 26.00, 25.83, 25.64, 22.70, 18.38, 14.13, -5.24. HR-MS (MALDI-TOF): calcd. for $C_{74}H_{132}N_2O_4S_2Si_2$ (M^+) 1232.9162; found: 1232.9154.

Synthesis of compound 4

Compound **3** (2.47 g, 2.00 mmol) was dissolved in 30 mL of chloroform, followed by the addition of *N*-bromosuccinimide (0.82 g, 4.60 mmol) at 0 °C. After stirred for 2 h, the mixture was extracted by dichloromethane and washed by saturated aqueous solution of $NaHCO_3$ for 3 times. After removal of solvents, the product was purified by silica gel column chromatography with petroleum ether (b.p. 60 - 90 °C) and dichloromethane (3:1, *v/v*) as eluents to give compound **4** as a red-violet solid (2.14 g, 76.9%). ^{1}H NMR (400 MHz, $CDCl_3$): δ = 8.63 (d, J = 4.0 Hz, 2H), 7.22 (d, J = 4.0 Hz, 2H), 3.92 (d, J = 8.0 Hz, 4H), 3.59 (t, J = 6.4 Hz, 4H), 1.87 (m, 2H), 1.53-1.46 (m, 4H), 1.29-1.21 (m, 76H), 0.90-0.86 (m, 24H), 0.04 (s, 12H). ^{13}C NMR (100 MHz, $CDCl_3$): δ = 161.40, 139.40, 135.31, 131.43, 131.19, 118.94, 108.04, 77.33, 77.22, 77.01, 76.70, 63.34, 46.36, 37.80, 32.92, 31.95, 31.24, 31.17, 30.03, 29.99, 29.72, 29.70, 29.67, 29.66, 29.61, 29.57, 29.48, 29.38, 26.21, 26.00, 25.83, 22.70, 18.38, 14.13, -5.24. HR-MS (MALDI-TOF): calcd. for $C_{74}H_{130}Br_2N_2O_4S_2Si_2$ (M^+) 1388.7372; found: 1388.7362.

Synthesis of compound 5

Compound **4** (2.14 g, 1.54 mmol) was dissolved in 30 mL of tetrahydrofuran, followed by the addition of hydrochloric acid (12 mmol/mL, 20 mL). The mixture was stirred at 80 °C for 2 h, extracted by dichloromethane and washed by saturated aqueous solution of $NaHCO_3$ for 3 times. After removal of solvents, the product was purified by silica gel column

chromatography with petroleum ether (b.p. 60 - 90 °C) and dichloromethane (1:2, *v/v*) as eluents to give compound **5** as a red-violet solid (1.58 g, 88.2%). ^{1}H NMR (400 MHz, $CDCl_3$): δ = 8.63 (d, *J* = 4.0 Hz, 2H), 7.22 (d, *J* = 4.0 Hz, 2H), 3.92 (d, *J* = 7.6 Hz 4H), 3.63 (t, *J* = 6.8 Hz, 4H), 1.87 (m, 2H), 1.59-1.52 (m, 6H), 1.29-1.21 (m, 76H), 0.89-0.86 (t, 6H). ^{13}C NMR (101 MHz, $CDCl_3$): δ = 161.41, 139.41, 135.33, 131.45, 131.16, 118.98, 108.01, 77.34, 77.02, 76.70, 63.09, 46.35, 37.76, 32.82, 31.95, 31.17, 30.00, 29.96, 29.72, 29.70, 29.68, 29.66, 29.59, 29.58, 29.54, 29.52, 29.43, 29.38, 26.18, 25.74, 22.71, 14.14. HR-MS (MALDI-TOF): calcd. for $C_{74}H_{115}Br_2N_2O_8S_4$ (M^+) 1469.5898; found: 1469.5893.

Synthesis of compound 6

Compound **5** (1.58 g, 1.54 mmol) was dissolved in 30 mL of dichloromethane, followed by the addition of pyridine and triethylamine. Then *p*-toluenesulfonyl chloride (5.92 g, 30.80 mmol) was added at 0 °C. The reaction mixture was stirred for 1 h and extracted by dichloromethane, then washed by dilute aqueous hydrochloric acid for 3 times. After removal of solvents, the product was purified by silica gel column chromatography with petroleum ether (b.p. 60 - 90 °C) and dichloromethane (1:1, *v/v*) as eluents to give compound **6** as a red-brown solid (1.41 g, 62.2%). ^{1}H NMR (400 MHz, $CDCl_3$): δ = 8.62 (d, *J* = 4.0 Hz, 2H), 7.79 (d, *J* = 8.4 Hz, 4H), 7.34 (d, *J* = 8.0 Hz, 4H), 7.21 (d, *J* = 4.0 Hz, 2H), 4.01 (t, *J* = 6.4 Hz, 4H), 3.92 (d, *J* = 7.6 Hz, 4H), 2.44 (s, 6H), 1.87 (m, 2H), 1.65-1.58 (m, 4H), 1.29-1.18 (m, 76H), 0.89-0.86 (t, 6H). ^{13}C NMR (100 MHz, $CDCl_3$) δ = 161.40, 144.58, 139.40, 135.29, 133.33, 131.45, 131.18, 129.79, 127.89, 118.97, 108.03, 77.34, 77.02, 76.70, 70.69, 46.35, 37.80, 31.95, 31.23, 31.17, 30.00, 29.72, 29.70, 29.68, 29.66, 29.57, 29.53, 29.50, 29.40, 29.38, 28.97, 28.85, 26.21, 25.36, 22.71, 21.64, 14.13. HR-MS (MALDI-TOF): calcd. for $C_{62}H_{102}Br_2N_2O_4S_2$ (M^+) 1160.5642; found: 1160.5641.

Synthesis of compound 7

Compound **6** (1.41 g, 0.96 mmol) was dissolved in 30 mL of *N, N*-dimethylformamide, followed by the addition of NaN_3 (0.25 g, 3.83 mmol) at room temperature. The reaction mixture was stirred for 7 h and extracted by dichloromethane, then washed by saturated aqueous solution of NaCl for 3 times. After removal of solvents, the product was purified by silica gel column chromatography with petroleum ether (b.p. 60 - 90 °C) and dichloromethane (2:1, *v/v*) as eluents to give compound **7** as a red-violet solid (0.581 g, 50.1%). ^{1}H NMR (400 MHz, $CDCl_3$): δ = 8.63 (d, *J* = 4.4 Hz, 2H), 7.22 (d, *J* = 4.0 Hz, 2H), 3.92 (d, *J* = 8.0 Hz, 4H), 3.25 (t, *J* = 6.8 Hz, 4H), 1.88 (m, 2H), 1.60-1.55(m, 4H), 1.30-1.21 (m, 76H), 0.88 (t, 6H). ^{13}C NMR (100 MHz, $CDCl_3$): δ = 161.40, 139.40, 135.32, 131.45, 131.18, 118.97, 108.02, 51.50, 46.35, 37.78, 31.95, 31.18, 30.00, 29.98, 29.72, 29.70, 29.68, 29.66, 29.57, 29.52, 29.49,

29.38, 29.17, 28.86, 26.74, 26.20, 26.18, 22.71, 14.14. HR-MS (MALDI-TOF): calcd. for $C_{62}H_{101}Br_2N_8O_2S_2$ (M^+) 1211.5850; found: 1211.5858. Elemental analysis: calcd. for $C_{62}H_{100}Br_2N_8O_2S_2$: C, 61.37; H, 8.31; N, 9.23; S, 5.28; found: C, 61.53; H, 8.32; N, 9.22; S, 5.31.

Synthesis of PDPP4T-N$_3$

Compound **7** (60.00 mg, 0.050 mmol), 5,5'-bis(trimethylstannyl)-2,2'-bithiophene (24.30 mg, 0.050 mmol) were added into a Schlenk tube with 5 mL of anhydrous toluene. The solution was degassed with nitrogen for 10 min. Then tris(dibenzylideneacetone) dipalladium (0) ($Pd_2(dba)_3$) (1.36 mg, 1.48 μmol) and tri(o-tolyl) phosphine (P(*o*-tol)$_3$) (1.55 mg, 5.93 μmol) were added in one portion and stirred for 1 h at 100 °C under nitrogen. After cooling to room temperature, the dark green solution was poured into methanol and the resulting precipitate was filtered. The polymer was purified by Soxhlet extraction using acetone, hexane sequentially to remove the oligomers and other impurities. Then, the chloroform fraction was precipitated in methanol, and filtrated. **PDPP4T-N$_3$** was obtained as a dark green solid (56.5 mg, 94 %). ^{1}H NMR (500 MHz, 1,1,2,2-tetrachloroethane-d_2, 373 k): δ = 8.75 (s, 2H), 7.24-6.98 (m, 7H), 3.95 (m, 4H), 3.13 (t, 4H), 1.91 (s, 2H), 1.48-1.18 (m, 80H), 0.81-0.78 (t, 6H). ^{13}C NMR (126 MHz, 1,1,2,2-tetrachloroethane-d_2, 373 k): δ = 51.92, 46.79, 38.16, 32.25, 31.85, 30.04, 29.63, 29.24, 28.85, 28.51, 26.76, 22.57, 14.03. Solid state ^{13}C NMR (100 MHz): δ = 160.85, 141.20, 136.94, 128.93, 124.22, 108.69, 45.73, 39.09, 32.84, 30.69, 23.59, 14.89. Elemental analysis: calcd. for $C_{70}H_{104}N_8O_2S_4$: C, 69.03; H, 8.61; N, 9.20; S, 10.53; found: C, 68.59; H, 8.47; N, 8.86; S, 10.41.

3. The Chemical structures of F$_4$BDOPV-2T and PDPP4T.

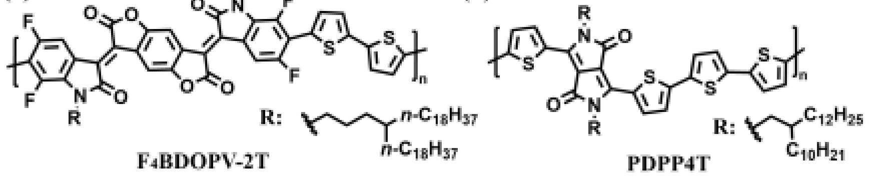

Figure S1. Chemical structures of **F$_4$BDOPV-2T** (a) and **PDPP4T** (b).

4. Gel Permeation Chromatography (GPC) of PDPP4T-N$_3$

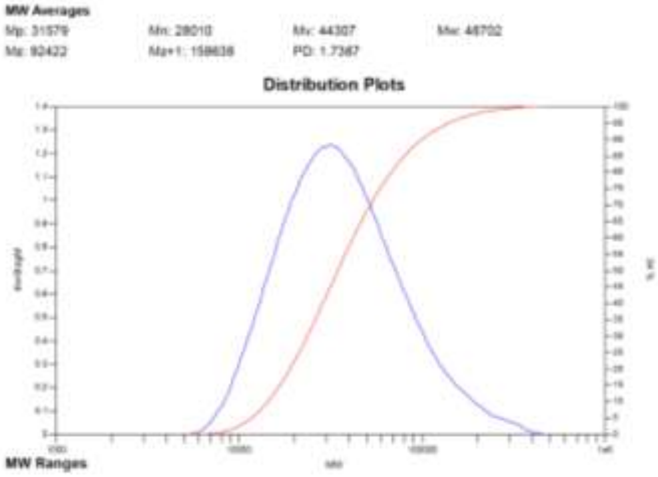

Figure S2. Gel Permeation Chromatography (GPC) of **PDPP4T-N$_3$**.

5. Absorption spectrum and cyclic voltammogram (CV) of PDPP4T-N$_3$

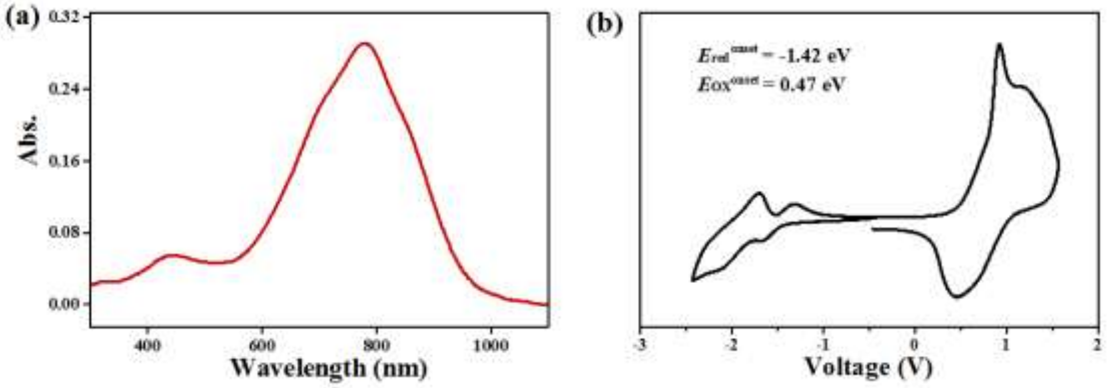

Figure S3. (a) Absorption spectrum of thin film of **PDPP4T-N$_3$**; (b) Cyclic voltammogram of **PDPP4T-N$_3$**.; the scan rate was 100 mV/s; potential vs. Fc/Fc$^+$.

6. FT-IR spectra

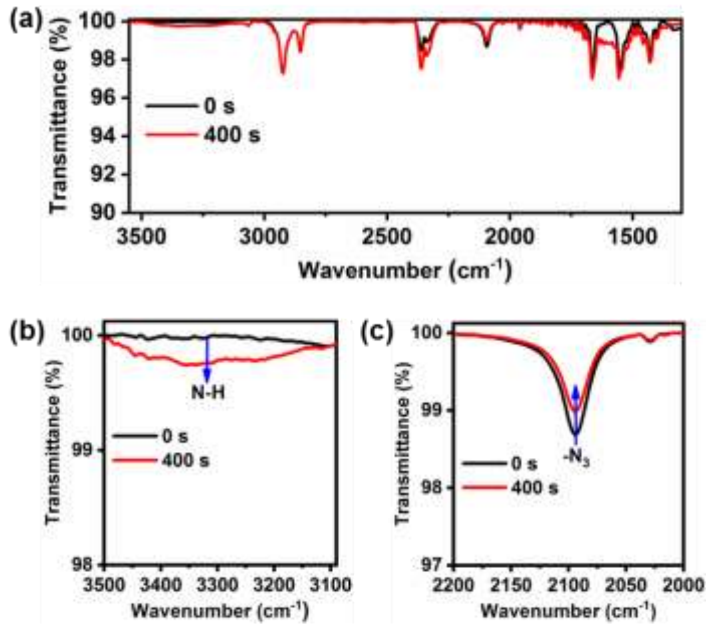

Figure S4. (a) The FT-IR spectra of the pristine thin film of **PDPP4T-N_3** and thin film after UV irradiation for 400 s; (b) the enlarged view of 3100-3500 cm^{-1}; (c) the enlarged view of 2000-2200 cm^{-1}.

7. Thermogravimetric analysis (TGA) of PDPP4T and PDPP4T-N_3

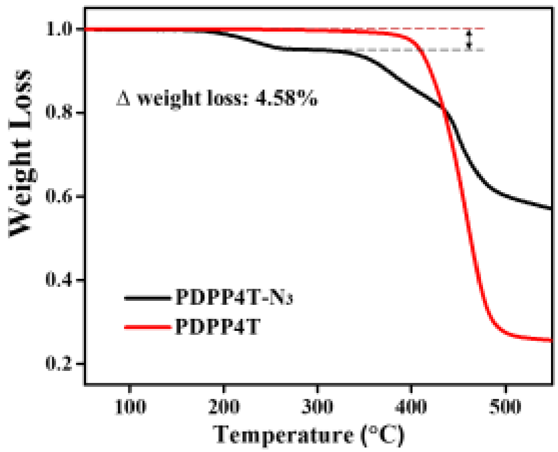

Figure S5. Thermogravimetric analysis curves of **PDPP4T-N_3 and PDPP4T**; Heating rate: 10 °C/min, from 50 °C to 550 °C under nitrogen atmosphere.

8. Parameters of the GIWAXS patterns

Table S1. Summary of the *q* values and the corresponding *d*-spacing of the (100) and (010) diffraction peaks.

	q value [$Å^{-1}$]		*d*-spacing [Å]	
	(100)	(010)	(100)	(010)
Pristine film	0.26	1.65	24.2	3.81
Film after UV irradiation	0.27	1.65	23.3	3.81

Chloroform-rinsed Film	0.27	1.65	23.3	3.81
Rinsed films after thermal annealing	0.27	1.65	23.3	3.81

9. Output curves of FETs with patterned thin films of PDPP4T-N_3

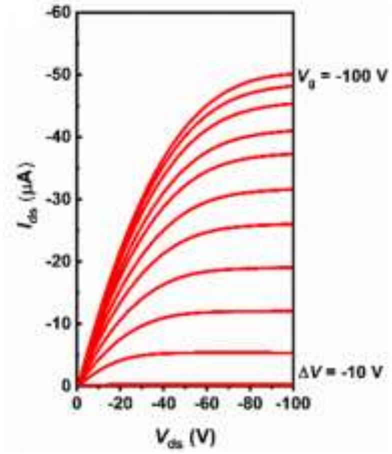

Figure S6. The representative output curve of FETs with patterned thin films of **PDPP4T-N_3**.

10. Performance data of FETs with pristine thin films of PDPP4T-N_3.

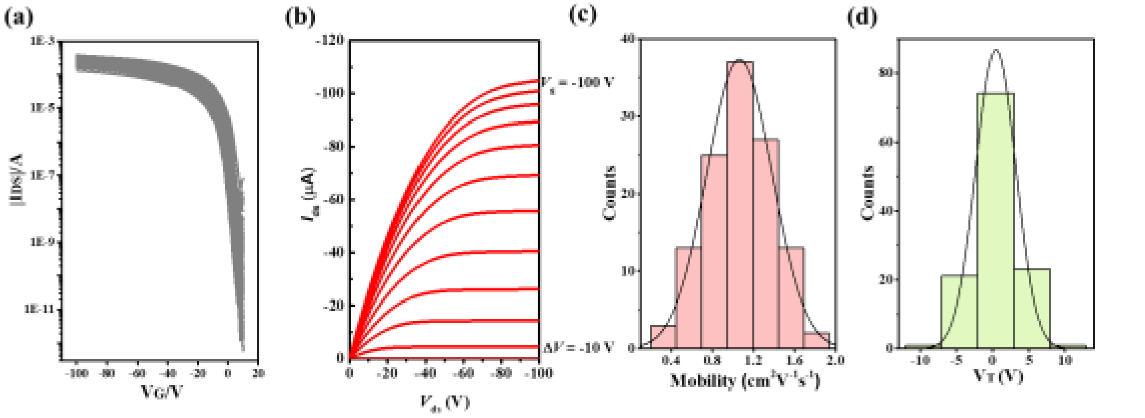

Figure S7. The performance data of FETs with pristine thin films of **PDPP4T-N_3**. (a) Transfer curves; (b) the representative output curve; (c) the distributions of mobilities; (d) the distributions of threshold voltages.

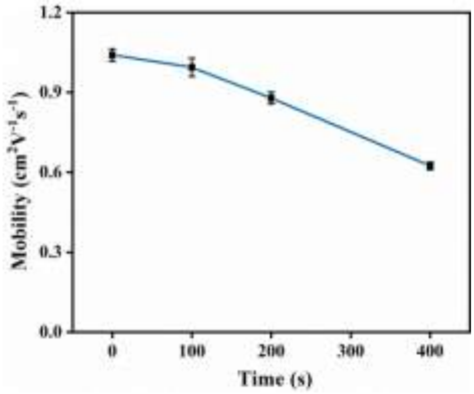

Figure S8. The variation of hole mobilities for devices with **PDPP4T-N$_3$** after UV irradiation (365 nm, 85 mW/cm^2) for different times. The average hole mobilities were based on five independent devices.

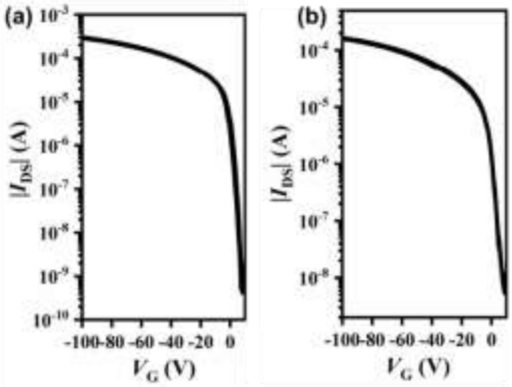

Figure S9. The representative transfer curves for the calculation of interfacial trap state density. (a) Five FET devices based on the pristine films; (b) Five FET devices based on the photo-patterned films.

Table S2. Summary of the charge mobility (μ), subthreshold swing (S) and interfacial trap state density (N_{int})[a] of five representative transfer curves of the pristine films and the cross-linked films.

	μ (cm^2 V^{-1} s^{-1})	S (V dec^{-1})	N_{int} (eV^{-1} cm^{-2})
Pristine films	1.13-1.43	1.58-1.91	1.77×10^{12}-2.15×10^{12}
Photo-patterned films	0.63-0.78	3.19-4.67	3.63×10^{12}-5.35×10^{12}

[a]the interfacial trap state density (N_{int}) of the FET devices was calculated according to the following equation $N_{\text{int}} = C_i[S\times q/(k_BT\ln10)-1]/q^2$, where C_i is the capacitance per unit area of the dielectric, q is the elementary charge, S is the subthreshold slope, k_B is the Boltzmann constant, and T is the absolute temperature.(S3)

11. Optical microscope image of inverter devices

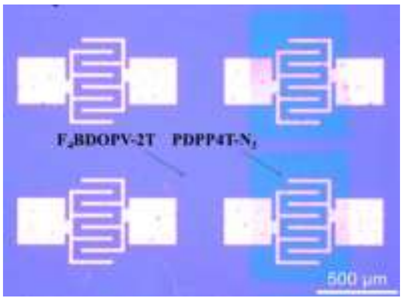

Figure S10. Optical microscope image of the patterned inverter devices; the *p*- and *n*-channel transistors in inverter device were connected by Au wire before measurement.

12. NMR spectra

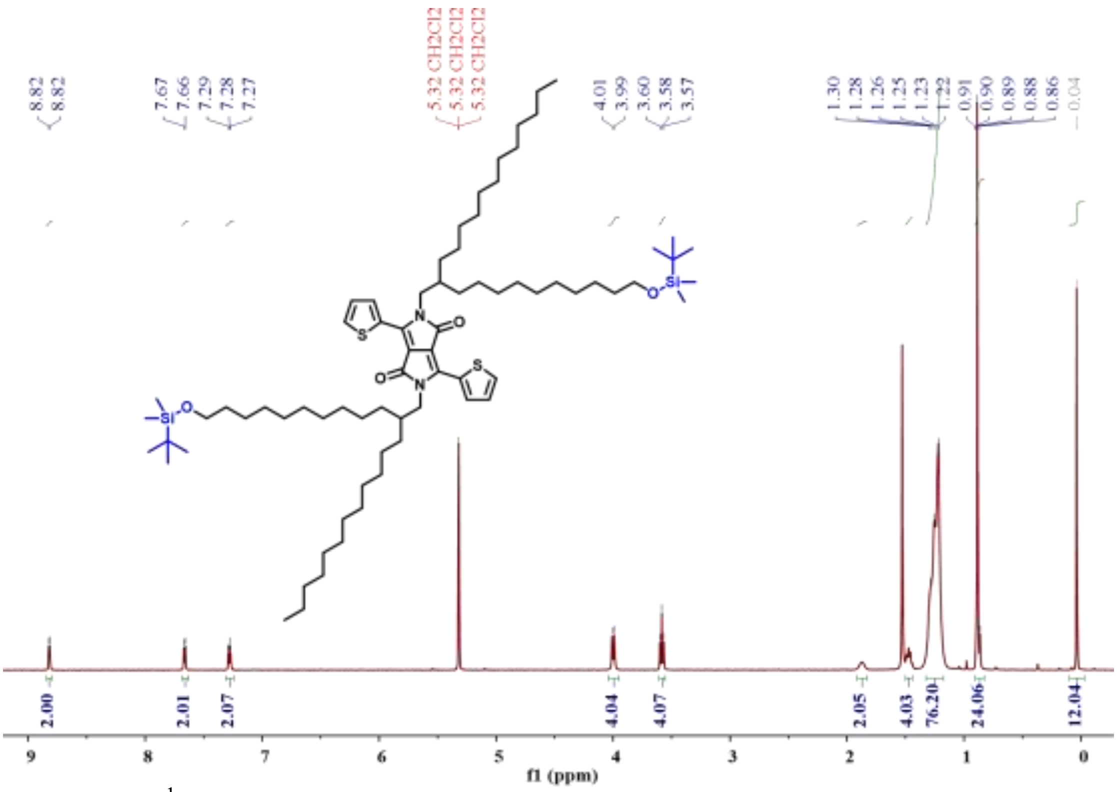

Figure S11. ^{1}H NMR spectrum of **3** in CD_2Cl_2.

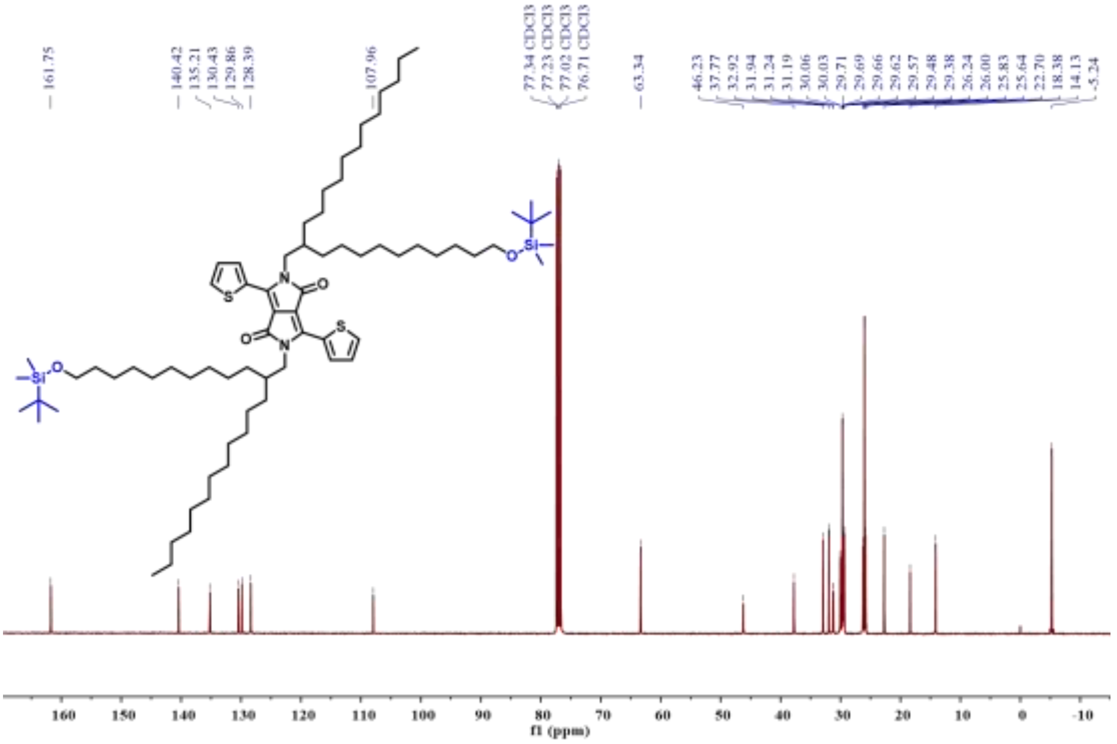

Figure S12. ^{13}C NMR spectrum of **3** in $CDCl_3$.

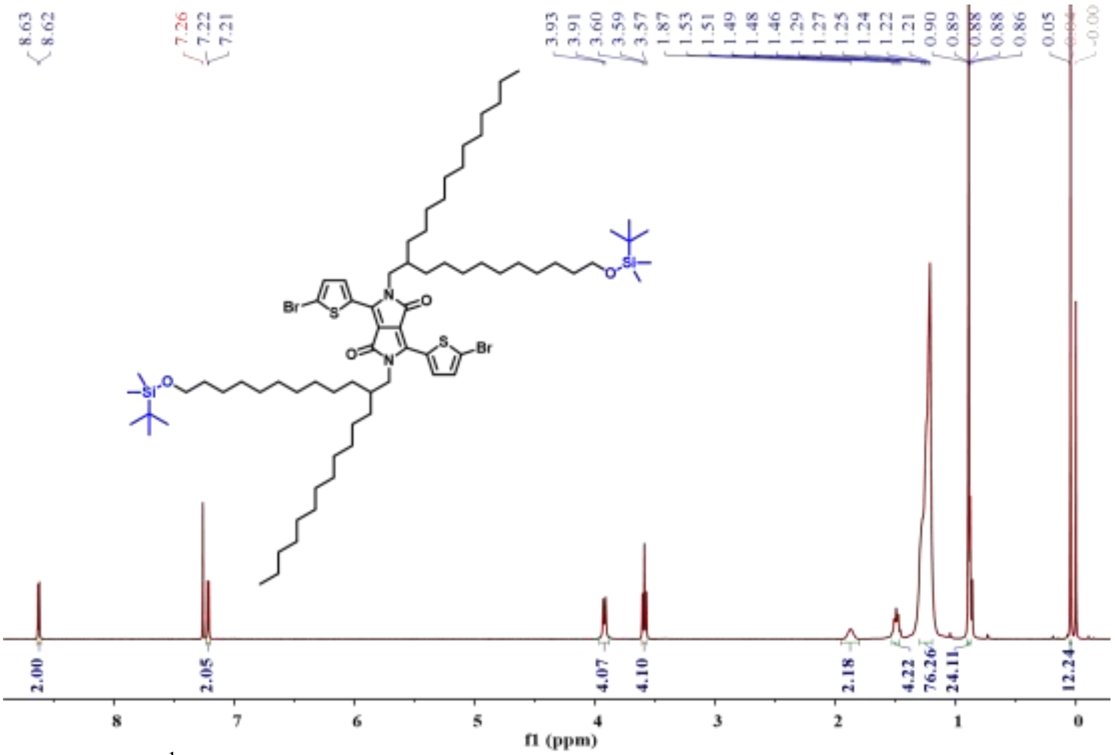

Figure S13. ^{1}H NMR spectrum of **4** in $CDCl_3$.

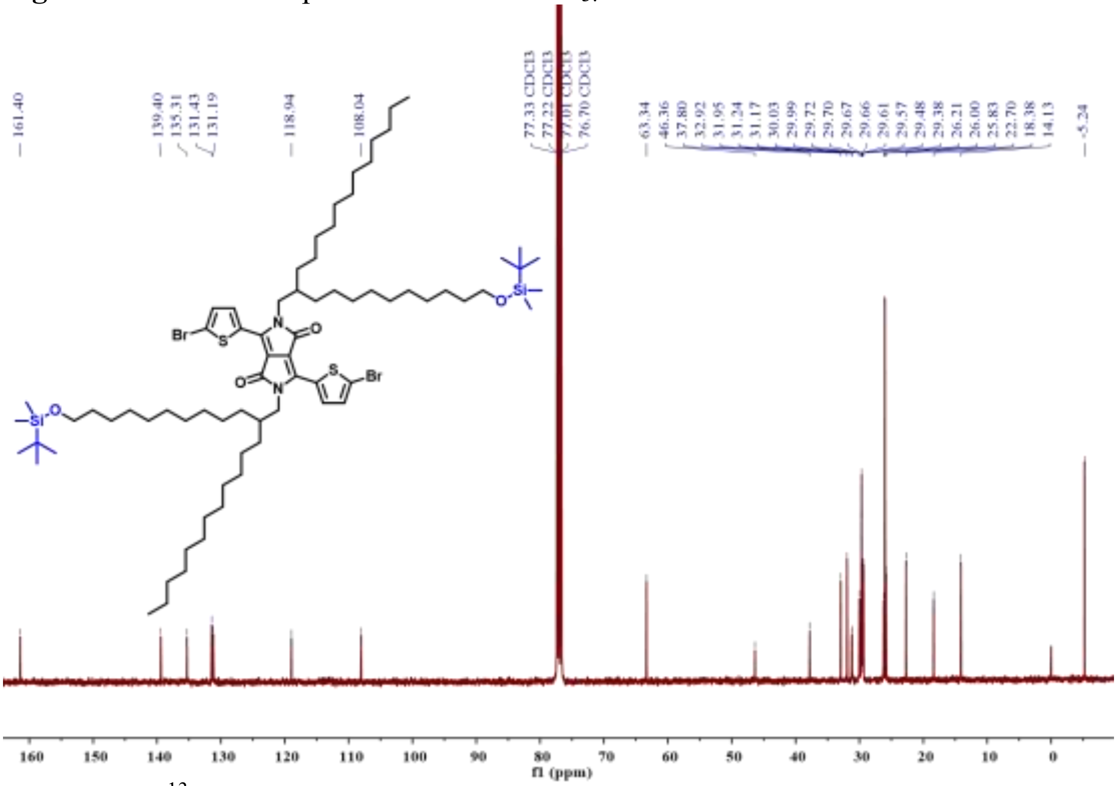

Figure S14. ^{13}C NMR spectrum of **4** in $CDCl_3$.

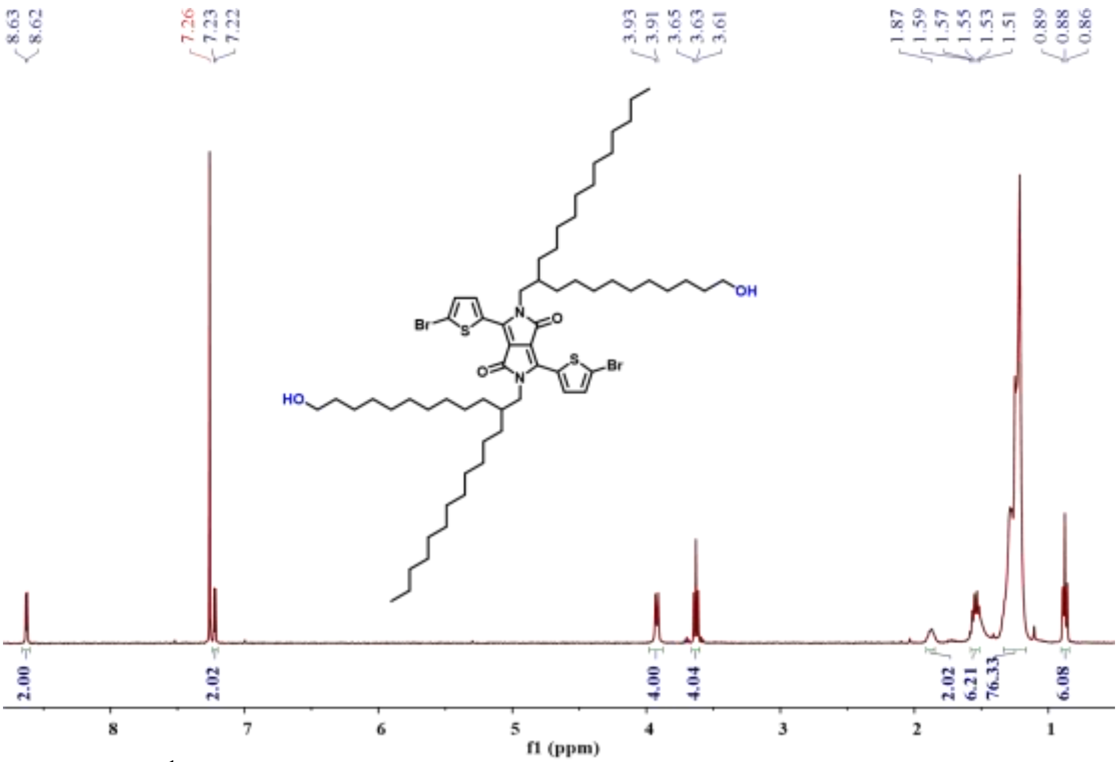

Figure S15. ^{1}H NMR spectrum of **5** in $CDCl_3$.

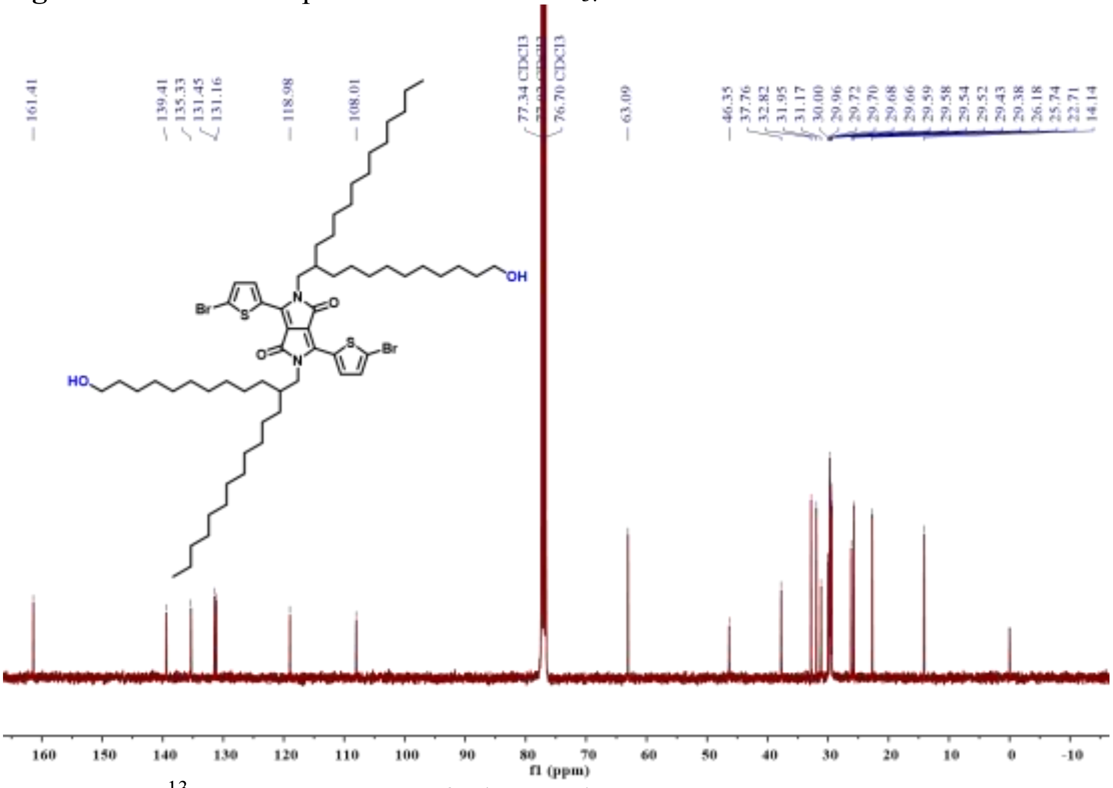

Figure S16. ^{13}C NMR spectrum of **5** in $CDCl_3$.

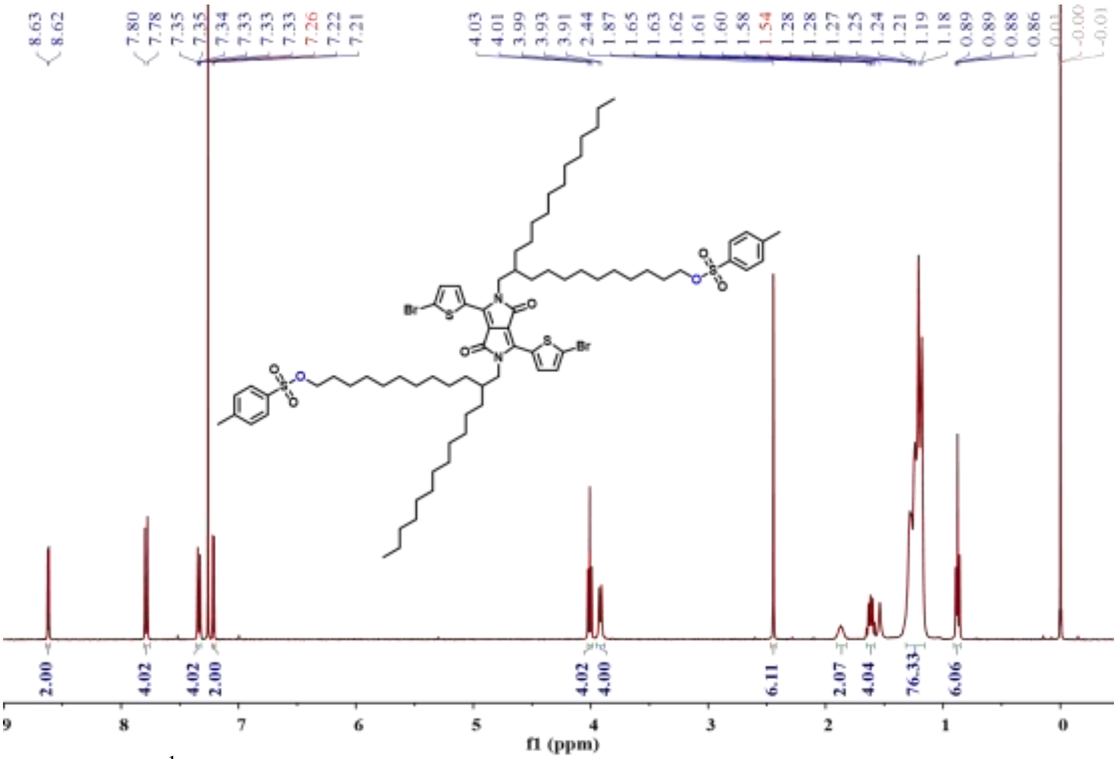

Figure S17. ^{1}H NMR spectrum of **6** in $CDCl_3$.

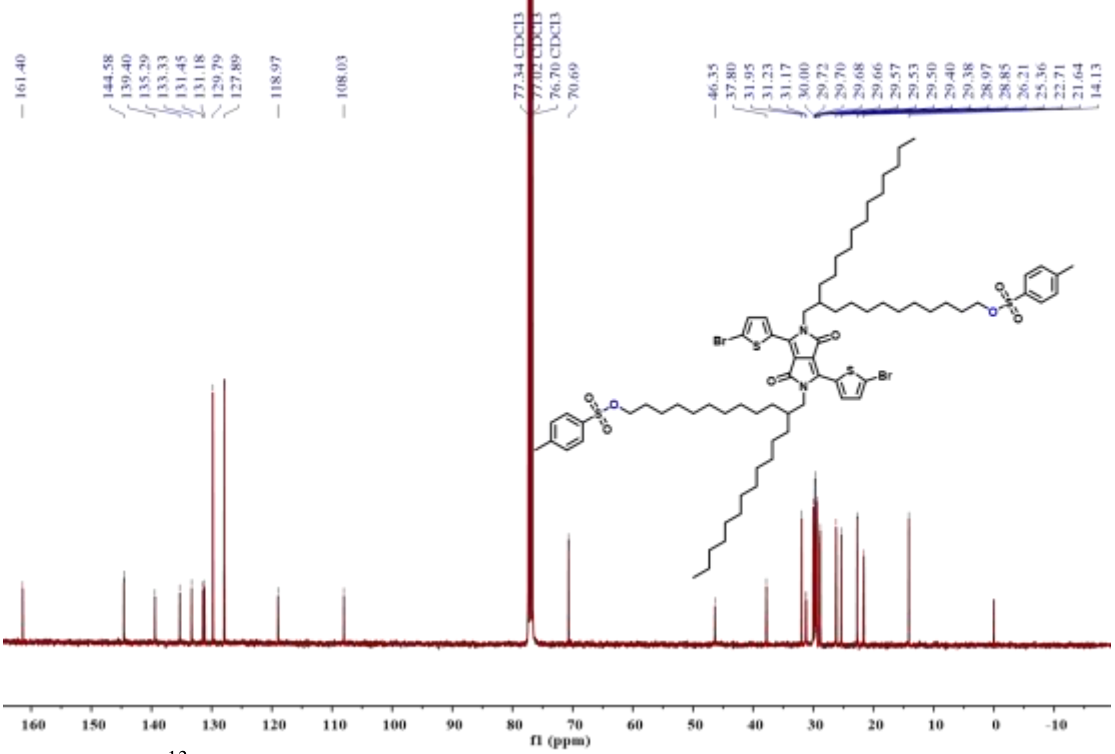

Figure S18. ^{13}C NMR spectrum of **6** in $CDCl_3$.

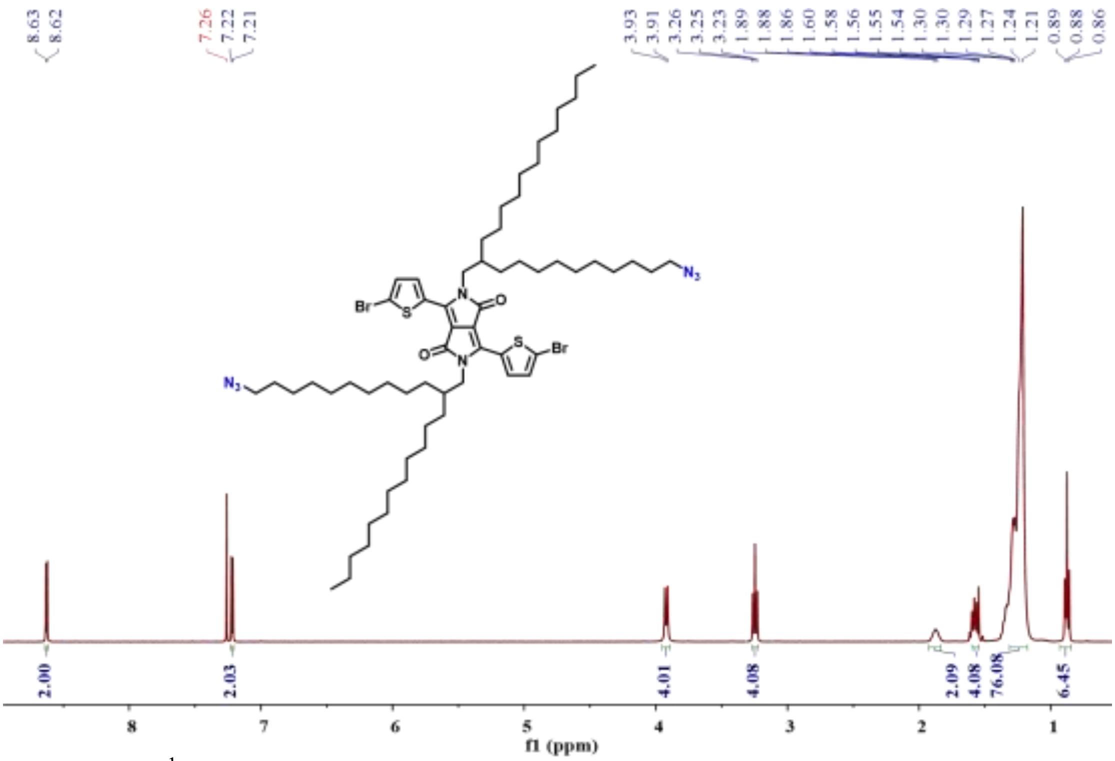

Figure S19. ^{1}H NMR spectrum of **7** in $CDCl_3$.

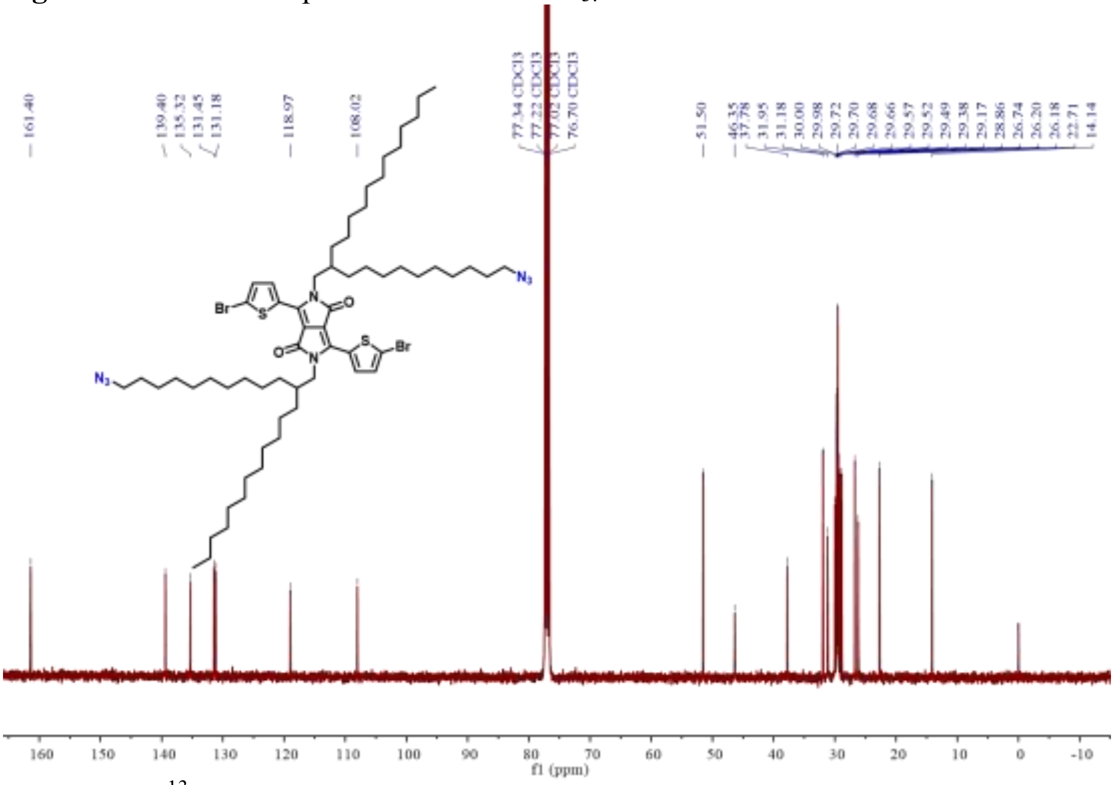

Figure S20. ^{13}C NMR spectrum of **7** in $CDCl_3$.

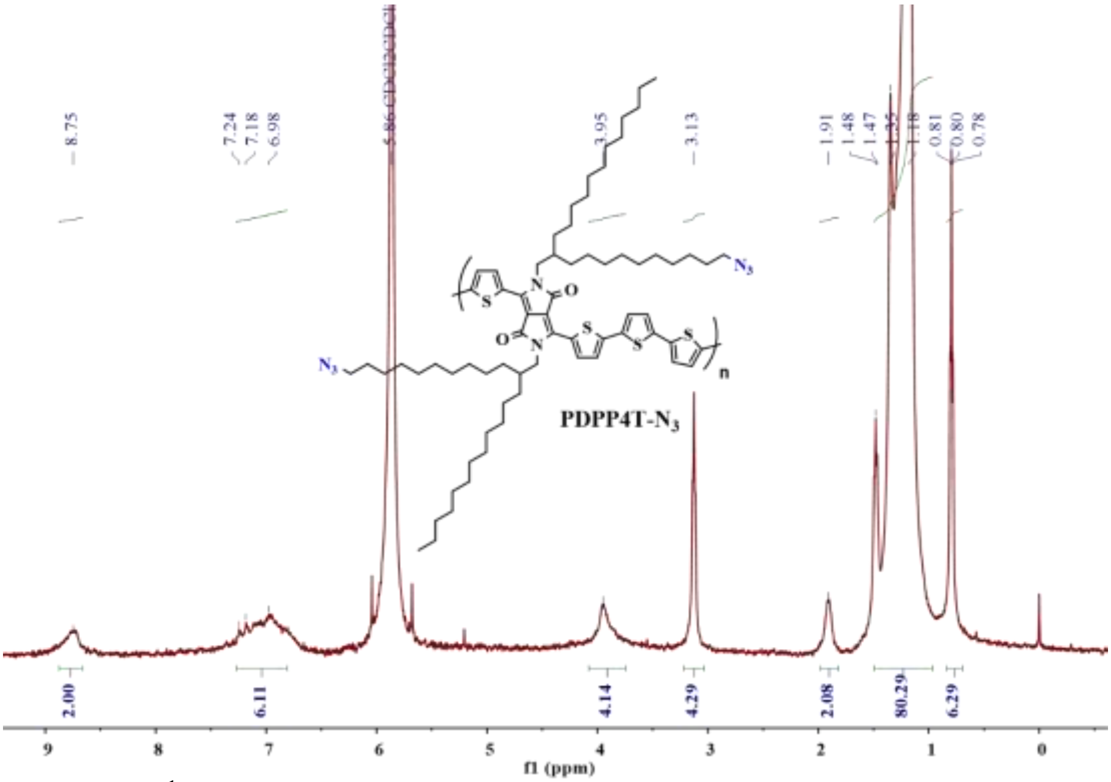

Figure S21. ^{1}H NMR spectrum of **PDPP4T-N$_3$** in 1,1,2,2-tetrachloroethane-d_2 at 373 K.

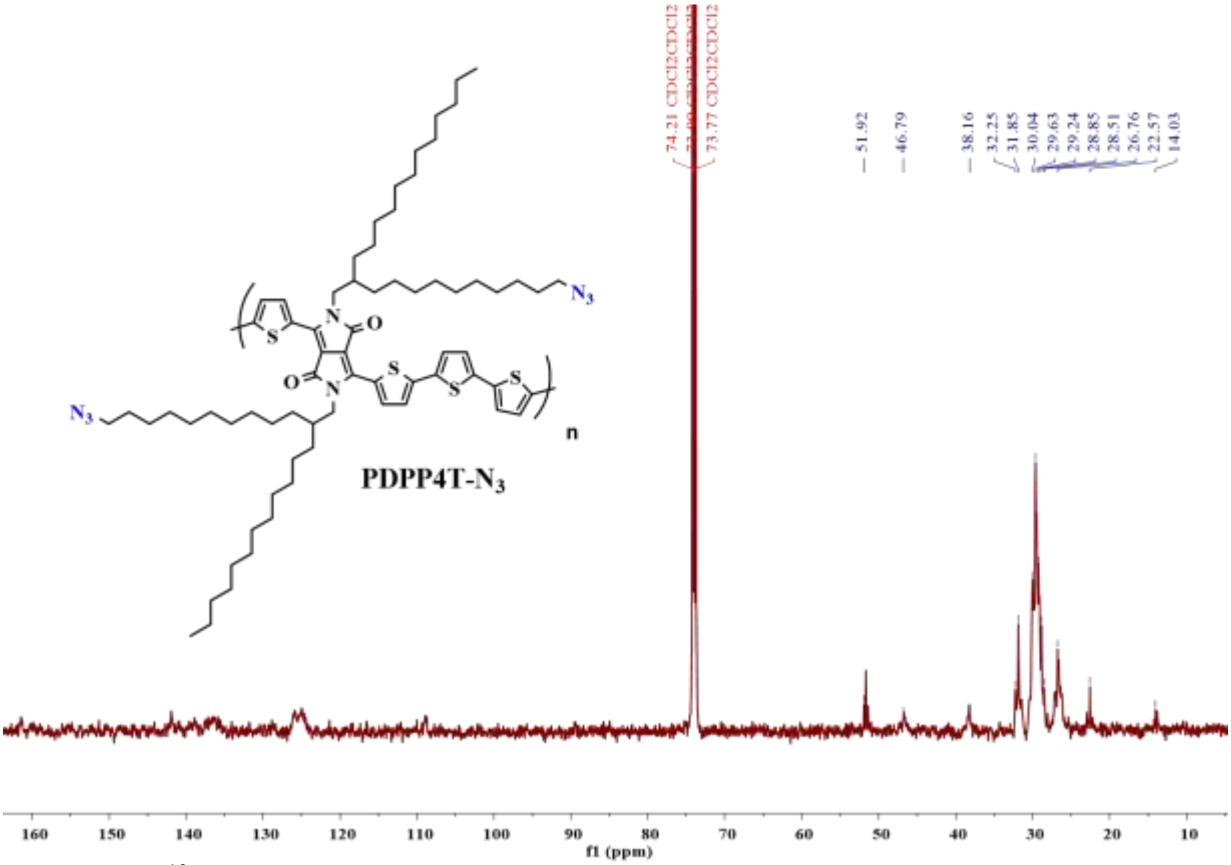

Figure S22. ^{13}C NMR spectrum of **PDPP4T-N$_3$** in 1,1,2,2-tetrachloroethane-d_2 at 373 K.

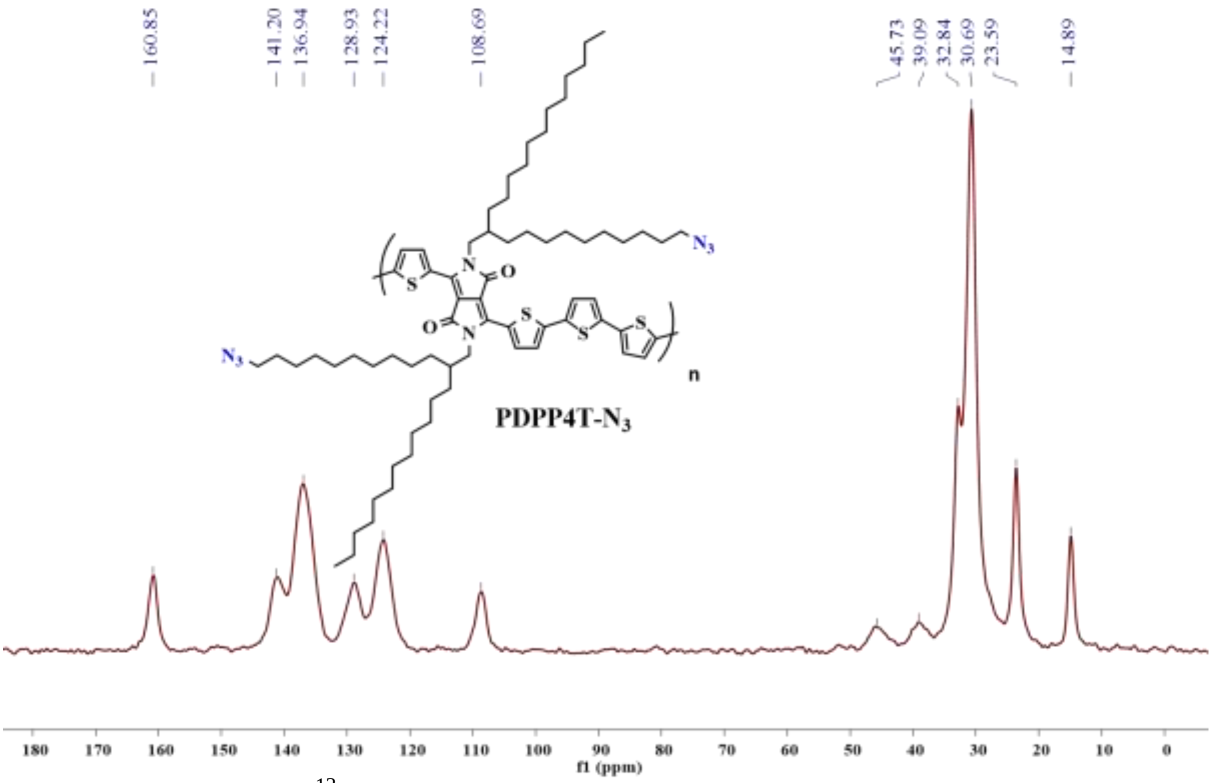

Figure S23. Solid state ^{13}C NMR spectrum of **PDPP4T-N$_3$**.

13. References

[S1] A. Zhang, C. Xiao, Y. Wu, C. Li, Y. Ji, L. Li, W. Hu, Z. Wang, W. Ma, W. Li, *Macromolecules* **2016**, *49*, 6431.

[S2] J. Tian, Z. Liu, W. Jiang, D. Shi, L. Chen, X. Zhang, G. Zhang, C. Di, D. Zhang, *Angew. Chem. Int. Ed.* **2020**, *59*, 13844.

[S3] W. L. Kalb, B. Batlogg, *Physical Review B* **2010**, *81*, 035327.
